# Book Reviews

**Published:** 1833-09

**Authors:** 


### BIBLIOGRAPHICAL NOTICES.

*The Principles and Practice of Obstetric Medicine, in a Series of Systematic Dissertations on Midwifery, and on the Diseases of Women and Children. Illustrated by numerous Plates.* By David D. Davis, M.D., M.R.S.L., Professor of Midwifery in the University of London. Parts XX and XXI. 4to. London: John Taylor.

While we freely admit that these parts of Dr. Davis's work contain a good deal of practical information, we still have to lament the existence of the fault to which we have before adverted, namely, a diffuse and inappropriate style of writing, which savours more of the efforts of a novel-writer to amuse his readers, than of a learned physician to instruct them. In the twentieth part, the subject of Hysteria is continued. We are far from denying that even simple cases of this very perplexing malady may require the use of the lancet; but ample experience convinces us that, in the very great majority of such cases, depletion must be had recourse to with the greatest caution. We cannot therefore agree with Dr. Davis as to the propriety of taking away from "twenty to five-and-thirty ounces of blood" in hysterical patients, even though they be "plethoric subjects," and the convulsive paroxysms very violent. To the abstraction of a much smaller quantity of blood we might perceive no objection, for the purpose of relieving any symptoms of determination of blood to the head; but we hold the doctrine to be quite unsound, (and the practice founded upon it fraught with mischief,) which teaches us that the "tremendous spasms" of hysteria are likely to be subdued by bloodletting. In no cases are these "spasms" more violent than in the persons of weak, delicate, and very pale-faced women, who would not bear the loss of blood at all, and much less of such a quantity as that mentioned by Dr. Davis. It is very true that he refers particularly to "plethoric" patients, but still the mode in which he states his opinion might lead the young and inexperienced practitioner to fly to the lancet as a general means of relieving the violent convulsive attacks which almost always occur in severe examples of hysteria, whatever may be the constitutional temperament or state of health of the patient. "Neglect of efficiently active practice (i. e. bleeding freely,) on the first accession of the disease, is no doubt a principal reason why the prognosis in hysteria is, as it actually is, so generally, and almost universally unfavorable."

Such is Dr. Davis's statement. Now, to us, the information is quite new that the prognosis in hysteria is "generally and almost universally unfavorable." We thought, and we think still, that the prognosis in hysteria is *almost always favorable*. In many cases there may be ample reason to fear that the disease may be tedious, and somewhat intractable, but the life of the patient is rarely in danger; and therefore the prognosis is as rarely unfavorable. We doubt indeed whether cases of simple hysteria ever end fatally. The patient may be long a sufferer, and may labour under the most complicated and apparently alarming symptoms; but, as a general fact, it certainly may be said that these symptoms disappear, more perhaps from the efforts of nature than the effects of the best directed medical skill, and the patient recovers her ordinary health and strength.

In cases of chronic hysteria, Dr. Davis recommends, and in our belief with propriety, the adoption of much the same line of treatment as in amenorrhœa. The moral management of such cases is also of the highest importance. The patient's mind should be occupied and amused. "Temporary change of residence; travelling; visiting attached and much respected friends; an extension of intercourse with cheerful and virtuous society; a constant succession of agreeable and useful employment; and, above all, daily exercise, preferably either on foot or on horseback, in the open air; will be found to furnish us with a pretty ample choice of materials for our moral prescriptions."

Upon the more formidable complications of hysteria Dr. Davis makes many very useful remarks, respecting both their nature and treatment.

Nymphomania is the next subject Dr. Davis discusses. In the parts of the work before us his comments upon this malady are not terminated.

---

*A Treatise on the Composition and Medical Properties of the Mineral Waters of Buxton, Matlock, Tunbridge Wells, Harrogate, Bath, Bristol, Cheltenham, Leamington, Malvern, Isle of Wight, Brighton, and the Beulah Spa, Norwood. With instructive Observations on the Drinking of the Waters, and the Use of the several Baths.* By Sir CHARLES SCUDAMORE, M.D., F.R.S. &c. Second Edition, corrected and much enlarged.—8vo. pp. 215. London: Longman and Co.

WE have no doubt but that this little work of Sir C. SCUDAMORE's will "take," both with the public and the profession. To the invalid who travels to any of our fashionable watering-places, and who is inclined to be his own patient, it will be very useful; and to the physician it will be equally so, as he is frequently asked to decide upon the real or supposed, the positive or comparative virtues of the mineral waters at the various places mentioned in the title-page. In 1819, the author visited several of the most remarkable watering-places in this country. The state of their mineral springs was the natural object of his inquiry, and he soon found reason to suspect the fidelity of the existing sources of authority respecting them. "Books were defective in describing the number of the springs in many places, and more or less erroneous as to the chemical properties of most of the waters." The additions to the present edition of the work consist of an account of the waters of Bristol, Brighton, and the Beulah Spa. Sir Charles was assisted in the chemical analysis of the different springs by Mr. GARDER and Mr. CHILDREN.

A brief geological sketch is given of the places he visited; the chemical analysis of each mineral water is then stated, and their medical properties are described.

We do not perceive that any point is omitted upon which the purchaser of such a work would seek for information. Sir Charles indulges in no exaggerated account of the superiority of one particular water over another, but assigns to each its fair and honest value: and this, we must observe, is a merit which works upon such topics rarely possess, especially when they proceed from the pen of some medical resident, who is of course very quick in discovering the great efficacy of the mineral springs of his own locality.

*The Teeth, in relation to Beauty, Voice, and Health: being the Result of Twenty Years' practical Experience and assiduous Study to produce the full Development and perfect Regularity of those essential Organs.* By John Nicholls, Surgeon-Dentist.—8vo. pp. 134. London: Hamilton, Adams, and Co.

This work is sufficiently free from technicality of expression to be read with interest by the public, for whom indeed we presume it to be especially intended; while at the same time the information it contains will furnish the medical student with many facts respecting the teeth which he ought to possess. It is divided into three chapters, and each chapter into three sections. In the first chapter many good practical remarks are made on the teeth in relation to beauty, voice, and health. In the second, the structure of the teeth; the temporary teeth; first dentition; the permanent teeth, and second dentition, are considered. The topics discussed in the third chapter are toothach, its cause, and proper treatment; anodyne cements; removal of decay; stopping the teeth; tooth-powders; and artificial teeth.

The style in which the work is got up does infinite credit to the publishers: we have never seen a more attractive looking medical volume.

